# Therapeutic Vaccination in Lung Cancer: Past Attempts, Current Approaches and Future Promises

**DOI:** 10.70322/jrbtm.2025.10010

**Published:** 2025-11-04

**Authors:** Samuel Patrick Young, Jie Sun

**Affiliations:** 1 Beirne B. Carter Center for Immunology Research, University of Virginia, Charlottesville, VA 22908, USA; 2 Division of Infectious Disease and International Health, Department of Medicine, University of Virginia, Charlottesville, VA 22908, USA; 3 Department of Microbiology, Immunology and Cancer Biology, University of Virginia, Charlottesville, VA 22908, USA

**Keywords:** Lung cancer, Therapeutic vaccine, Mucosal vaccination

## Abstract

Lung cancer represents a significant burden on global health, necessitating the need for new and effective treatment strategies that expand our current therapeutic repertoire. Immunotherapy, namely immune checkpoint blockade (ICB), has revolutionized lung cancer therapy over the last decade by invigorating anti-tumor T cell responses to prolong survival and quality of life. However, not all patients benefit from ICB, emphasizing the need for novel immunotherapeutic strategies that engage other immune functionalities to offer synergy with already available therapies. There has been a longstanding interest in deploying lung cancer vaccines to generate or enhance tumor antigen-specific T cell responses for greater tumor control. Thus far, success has been limited to early-stage clinical trials, where safety, generation of antigen-specific T cell responses in blood sampling, and some patient benefits have been established. Moving forward, the establishment of widespread clinical success in large-scale trials is a necessity to bring lung cancer vaccines into the therapeutic arsenal. In this review, we examine the logic and mechanisms behind therapeutic lung cancer vaccines, before critically and iteratively examining past and current attempts in lung cancer vaccinology. We also look at early pre-clinical studies and outline the future for therapeutic lung cancer vaccines.

## 1. Introduction

Lung cancer is among the deadliest cancers worldwide, with around 2 million new cases and 1.8 million deaths annually [1]. This high mortality is partly attributable to the significant metastatic potential and mutational heterogeneity culminating in complex, diffuse, multi-tissue pathology [2–6]. Additionally, lung cancer typically has a late symptomatic onset and screening detection, and most initial detections of lung cancer are made at an advanced stage [7,8]. The World Health Organization (WHO) categorizes lung cancer into two broad categories primarily based on histological cellular morphology: non-small cell lung cancer (NSCLC, comprising ~85% of all cases) and small-cell lung cancer (~15%) [9]. Traditional lung cancer treatment options include surgery, radiation, and chemotherapy; however, such strategies only offer transient benefits that fail to prevent eventual relapse [10]. Contemporary therapeutic methods aim to synergize with traditional treatment modalities in two major ways: (i) through targeted inhibition of the essential molecular pathways driving cancer cell growth and survival (targeted therapy), or (ii) by therapeutic modulation of the ability for the immune system to persistently recognize and kill cancer cells (immunotherapy) [10–12]. The use of targeted therapies in combination with chemotherapy applies to patients harboring targetable oncogenic mutations and has demonstrated improved progression-free (PF) survival and overall survival (OS) [10,11]. However, targeted therapy requires an actionable molecular target to be expressed by cancer cells, which many patient tumors lack or develop resistance to following treatment [10–12].

The last decade has seen great advances in lung cancer immunotherapy, drugs designed to reinvigorate insufficient anti-tumor immunity [13]. Immunotherapeutic efforts thus far have particularly emphasized the enhancement of cytotoxic T-cell (CTL) mediated anti-tumor immune responses to tumor-specific antigens (TSA) and/or tumor-associated antigens (TAA) [13–15]. There are a variety of clinically approved and experimental methods being explored, including monoclonal antibodies designed to inactivate immunosuppressive pathways inhibiting T cell activation and recognition of cancer cells (Immune Checkpoint Blockade, (ICB)), bispecific T cell-engaging antibodies (BiTEs), adoptive cellular therapies, immune modulating cytokines, and therapeutic vaccines [16–21]. ICB has become preeminent in lung cancer care, particularly for NSCLC, as a frontline, neoadjuvant, or adjuvant therapy alone or in combination with other therapeutic strategies [16,17,22–24]. Despite these successes, the universal efficacy and curative capacity of ICB is often constrained by factors including a lack of T cell infiltration into the TME, downregulation of Major Histocompatibility Complex I (MHC)-antigen presentation to T cells, and/or upregulation of alternative immune checkpoint pathways [25,26].

Considering the demonstrated potential of immunotherapy to treat subsets of advanced-stage NSCLCs, the timely development of novel immunotherapeutic strategies beyond ICB is imperative to integrate other functionalities of the immune system into clinical care. Historically, lung cancer vaccines have demonstrated limited efficacy, potentially resulting from a variety of factors, including a generally low tumor mutational burden (TMB), extensive heterogeneity in antigen expression and function, inadequate vaccine formulation or delivery, as well as existing or acquired mechanisms of immune escape among patient cohorts [21,27]. However, revisitation of therapeutic lung cancer vaccines has been spurred by the remarkable successes of mRNA- and peptide-lipid nanoparticle (LNP) vaccines as a rapidly deployable and effective measure against infectious diseases, particularly SARS-CoV-2 [28,29].

In this review, we will establish the important immune mechanisms and cell types responsible for conferring therapeutic cancer vaccine efficacy, before systematically and iteratively examining past and current therapeutic vaccination attempts and approaches in lung cancer. We will then look at recent pre-clinical studies to understand and explore future directions to guide the next generation of lung cancer vaccines.

## 2. Cellular and Molecular Mechanisms of Therapeutic Vaccination in Lung Cancer

**Figure 1.**
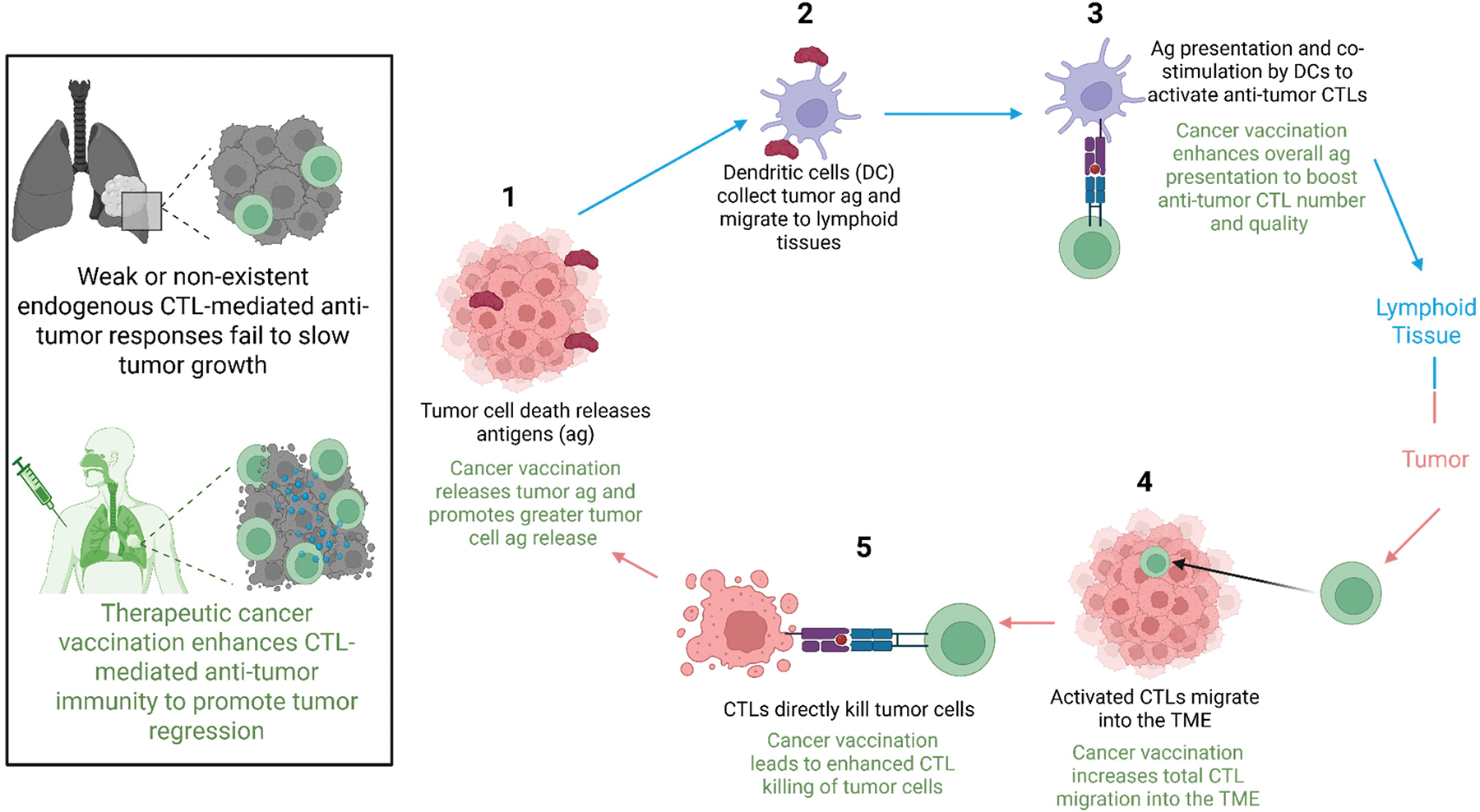
Mechanisms of CTL-mediated anti-tumor immunity and how cancer vaccination contributes to enhancement of this process. Cancer vaccination promotes enhanced tumor antigen (ag) release (1), resulting in uptake of ag by dendritic cells (2) and increased ag presentation in lymphoid tissues to generate anti-tumor CTLs (3), that migrate into the tumor microenvironment (TME) (4) to promote greater tumor cell killing (5) and further drive this overall process.

Cancer vaccines are designed to elicit highly specific, robust immune activation against selected TSAs or TAAs to heighten B and T cell activation and enhanced antigen-specific effector functionality (Figure 1) [21,27]. Prophylactic vaccination aims to confer long-term disease protection through the generation of antibodies and establishment of memory T and B cell populations that elicit rapid immune responses upon antigen re-encounter [30]. In contrast, therapeutic cancer vaccination primarily aims to boost CTL responses to promote the killing of already established tumors by inducing immediate CTL expansion and anti-tumor activity that is sustained through long-lasting memory CTL generation [27,30,31].

Boosting CTL-mediated anti-tumor immunity through therapeutic vaccination is mechanistically driven by amplifying tumor antigen uptake by dendritic cells (DCs) [32,33]. CTLs recognize tumor antigens on the DC surface as fragmented peptides loaded onto major histocompatibility complex (MHC) class I molecules in a process called antigen presentation [34–37]. This peptide: MHC I complex is recognized by CD8+ T cells through their T cell receptor (TCR) upon direct engagement, forming an immunological synapse and is the first of two signals required for T cell activation [38]. Oftentimes, cancer vaccines include adjuvants, such as granulocyte-macrophage colony-stimulating factor (GM-CSF) or toll-like receptor (TLR) agonists, to specifically increase DC function and antigen presentation efficiency [38].

Antigen uptake following vaccination takes place in peripheral tissues where DCs reside, resulting in the production of chemokines, such as CCL19 and CCL21, to guide the migration of DCs from the afferent lymphatics into lymphoid tissues, typically the tumor draining lymph nodes (tdLN), to initiate antigen presentation [39–41]. However, transient development of lymphoid tissues within the lung, termed inducible bronchus associated lymphoid tissue (iBALT) has been observed in all stages of NSCLC patients, correlating to a favorable outcome, though the exact processes driving the formation and maintenance of these structures and their anti-tumor benefits are unclear in the context of lung cancer [42–44]. It also remains unclear whether vaccination alone can induce iBALT formation or enhance its potential anti-tumor function. Further exploration into the mechanisms driving lung cancer-associated iBALT formation—such as local antigen availability and cytokine cues required for its establishment and maintenance—could address this possibility.

Upon peptide: MHC I recognition by CD8+ T cells, additional co-stimulatory interactions with DCs provide the second signal necessary for full T cell activation by promoting survival, proliferation, and migration to cancerous tissues [45–48]. CD8+ T cells then interpret external biomolecular cues from their environment, including cytokines and metabolites, to fully establish as CTLs capable of direct tumor cell killing upon cognate peptide: MHC I recognition [45,49–51]. CTLs are also potent sources of pro-inflammatory cytokines, such as IFN-γ and TNF, capable of invigorating a more inflammatory TME, an especially important consideration for many lung TME that are immunosuppressive in nature [45,50,51].

The benefits of therapeutic cancer vaccination are not exclusively limited to CTL function. The presence of CD4+ T helper cells in NSCLC has been correlated with increased OS and response to immunotherapy [52,53]. CD4+ T cells are activated through MHC class II presentation of peptide antigen by DCs and play a key supportive role in coordinating and optimizing anti-tumor CTL responses [53,54]. CD4+ T cells aid the anti-tumor CTL response primarily through their help to DCs for better CTL priming or production of cytokines, including IL-2 and IFN-γ to promote CTL survival, proliferation, and/or effector function [54]. CD4+ T cells have also been shown to exert tumoricidal activity, albeit to a lesser degree than CTLs [54,55]. B cells can also provide benefits following therapeutic cancer vaccination through their B cell receptor (BCR), individually capable of recognizing protein antigens [56]. Given B cells can make up a significant portion of the lung TME, generation of antibody responses against tumor antigen may play a significant role in tumoricidal activity through antibody-dependent cellular cytotoxicity (ADCC), an immune killing mechanism by which a target cell expressing antigen is coated in antibodies containing an Fc region recognizable to innate immune cells that deliver a cytotoxic payload to lyse the cell [57–59].

## 3. Vaccination Delivery Platforms Differentially Shape Immune Response to Lung Cancer

Considering the mechanisms and cell types responsible for therapeutic vaccination efficacy, the delivery system and antigen formulation play a key role in efficiently co-opting the anti-tumor immune response. Common cancer vaccine delivery types include recombinant peptide, protein, nucleic acid (DNA or RNA), whole tumor cell (WTC), and antigen-loaded dendritic cells (DC). Macromolecular vaccines, including peptide, protein, DNA, and RNA, require additional encapsulation and modification to ensure deposition into tissue and/or modifications to temper overt inflammatory responses caused by innate immune detection [60–62].

WTC vaccines use live or lysed tumor cells from donors that are infused with immunostimulants into a recipient patient, providing a broad range of antigens for the recipient immune system to process and respond against [63]. On the other hand, DC vaccines utilize patient-derived DCs that are activated by loading them with pre-selected tumor antigens *ex vivo* before reinfusion back into the patient [64].

In peptide-based vaccine approaches, tumor antigenic peptides are pre-selected for their optimal immunogenicity, packaged, and combined with adjuvants to promote efficient uptake and presentation by DCs on MHC molecules [65]. This vaccine approach relies particularly upon the uptake of exogenous protein antigens by DCs in a process called cross-presentation [66]. DNA and RNA vaccine approaches rely on endogenous translation of protein antigens within host cells, followed by intracellular processing into an antigenic peptide repertoire [67,68]. This process yields a broader, more diverse spectrum of antigens for presentation, though immunogenicity of each individual peptide may not be maximal [67,68].

Beyond the formulation or delivery mechanism of tumor antigens, the choice of antigenic target(s) is equally important. TSAs represent the most targeted class of tumor antigens, as they are exclusively expressed by tumor cells [69,70]. In lung cancer, TSAs include somatically mutated oncogenes responsible for driving lung tumor growth, the most common mutations occurring in genes such as *EGFR*, *KRAS,* and *ALK* [71–74]. Somatic mutations also give rise to a class of heterogeneous TSAs, termed neoantigens [70,75,76]. Unlike oncoproteins, neoantigens are not essential for cancer cell growth and survival, yet can elicit strong anti-tumor immune responses and are unique to individual tumors [76].

TAAs, on the other hand, are not uniquely expressed by tumor cells, but are either overexpressed or post-translationally modified by tumor cells to allow for preferential immune recognition and killing [77,78]. Germline oncogenic mutations, cancer testis antigens (CTAs), chromosomal fusion proteins, and glycosylated proteins represent major sources of TAAs [79]. Advantages to using TAAs for cancer vaccine delivery surround their demonstrated immunogenicity, ability for mass production, and usage in patient’s pre-screened for specific TAAs [79,80].

## 4. Lung Tumor Immune Evasion Mechanisms Confound Vaccine Efficacy

Despite a clear rationale for the benefit of therapeutic lung cancer vaccination, there is no clinically approved therapeutic lung cancer vaccine. Inefficient antigen delivery or inadequate antigen selection at a larger scale could partially explain why clinical implementation has been elusive. However, mechanisms of extrinsic and intrinsic tumor immune evasion prior to or following vaccination also need to be considered. Lung cancer cell intrinsic mechanisms of immune evasion surround the limitation of antigen recognition by T cells [81,82]. One such mechanism is through increasing expression of PD-L1/2, the cognate ligands for the surface receptor PD-1 expressed by T cells to antagonize TCR signaling [83,84]. Other major sources of tumor cell intrinsic immune evasion include chronic antigen persistence, loss of MHC I expression, and secretion of immunosuppressive cytokines [26,85–88].

Waning or cessation of vaccine-induced immunological memory is another key factor confounding lung cancer vaccine efficacy [89]. While vaccination drives immediate T cell activation and proliferation via antigen deposition, maintaining sustained T cell responses occurs in part through vaccine-induced, long-lived memory T cell populations present in circulation and/or local tissues [90–93]. Optimization of vaccine formulations, adjuvants, and booster schedules helps to achieve durable T cell immunity that can persist long-term [94,95]. However, tumor escape resulting in loss or downregulation of vaccine-encoded tumor antigen expression or MHC presentation by tumor cells can potentially reduce anti-tumor durability and booster efficacy [96–98]. Additionally, inadequate breadth and persistence of long-term vaccine-induced immunity could select for tumor cell clones lacking target antigens, ultimately failing to control tumor growth, resulting in eventual relapse [99–101].

Extrinsic forms of immune evasion surround the constituents of the TME and vasculature at large. Production of pro-inflammatory cytokine IL-2 acts to strengthen CTL responses; however, they also give rise to regulatory T cells (Tregs) [102–104]. Tregs are a CD4+ T cell subset that acts to dampen immune responses through the production of anti-inflammatory cytokines such as transforming growth factor beta (TGF-β) and IL-10, or through the inhibition of DC maturation and function [102–106]. An impermeable TME is another immune evasion mechanism, by which stromal cells, including cancer associated fibroblasts (CAFs) can produce rigid extracellular matrix to limit immune cell entry and proximal interactions with tumor cells [81,82,107,108]. Tumor associated endothelial cells (TECs) also promote immune evasion by controlling nutrient adsorption and immune infiltration into the TME, creating a largely hypoxic environment unfavorable for T cell activation and function [109].

Altogether, while therapeutic lung cancer vaccines make sense mechanistically as an effective method of tumor control, clinical success has been scarce. In fact, only one cancer vaccine has garnered FDA approval, PROVENGE^®^ (sipuleucel-T), an autologous DC vaccine for prostate cancer [110,111]. Underlying a lack of clinical success is suboptimal antigen delivery into tumor or tumor adjacent tissues, as well as copious tumor immune escape mechanisms [60,81,82]. To better prepare future vaccination attempts, we will begin by reviewing previous key clinical trials that have shaped present-day clinical trials.

## 5. Past Therapeutic Vaccination Attempts in Lung Cancer

### 5.1. Peptide and Protein Vaccines

#### 5.1.1. MAGEA3

MAGEA3 is a cancer testis antigen (CTA), a class of TAAs that are endogenously expressed in immune-privileged sites [112,113]. However, tumor cells can aberrantly upregulate MAGEA3 in non-immune privileged tissues, triggering an anti-tumor immune response [114,115]. MAGEA3 expression is detected in over 30% of NSCLC patients, positively correlating with smoking status, advanced tumor stage, and poor outcome [116–118]. Phase I and II clinical trials of MAGEA3 peptide-based vaccines in patients with completely resected stage IB-II NSCLC demonstrated safety and showed a trend toward improved disease-free interval (DFI) and disease-free survival (DFS) [119,120]. However, expansion into the Phase III trial, MAGRIT, revealed no significant increase in DFS and overall survival (OS) compared to placebo, halting further investigation [121,122]. This disappointing result had further ripple effects, contributing towards the shutdown of a Phase II clinical trial targeting another aberrantly expressed CTA, preferentially expressed antigen in melanoma (PRAME), despite positive safety and immunogenicity results [123].

#### 5.1.2. EGF

CIMAvax-EGF is a protein-protein conjugate vaccine adjuvanted with an O/W emulsifier, Montanide ISA 51 [124]. CIMAvax-EGF is a conjugate of recombinant epidermal growth factor (EGF) with P64, a recombinant carrier protein derived from *Neisseria meningitides,* acting as a foreign antigen to heighten overall immunogenicity [124,125]. Unlike many cancer vaccines designed to elicit potent CTL responses, CIMAvax-EGF intends to starve cancer cells overexpressing epidermal growth factor receptor (EGFR) through the generation of neutralizing antibodies against its cognate ligand, EGF [125]. In Cuba, CIMAvax-EGF is available for use as a maintenance therapy following progression through Phase IV clinical trials in the late 2010s, where patients with advanced NSCLC receiving prior chemotherapy were enrolled, demonstrating safety, reduced EGF in serum, and increased OS [126]. A positive correlation between CD4+ and CD8+ T cell presence following chemotherapy and greater OS following vaccination was also found [127]. Most recently, a phase III clinical trial in Cuba investigated the use of CIMAvax-EGF as a switch maintenance therapy in patients achieving stable disease after receiving first-line therapy and found disease control in 36.8% and 19.8% at 6- and 12-month post-vaccination, respectively [128]. CIMAvax-EGF is not yet approved in the U.S.. However, Phase I and II trials in the U.S. have suggested that combinatorial therapy of CIMAvax-EGF with ICB (Nivolumab) further enhances the antibody response to EGF compared to vaccination alone [129,130]. However, the current focus of U.S. based CIMAvax-EGF trials is towards its use as a prophylactic vaccine for patients at risk for developing EGFR-driven NSCLC (NCT04298606).

#### 5.1.3. MUC1

Mucin-1 (MUC1) is a membrane-bound glycoprotein expressed on the apical surface of lung epithelial cells [131–133]. MUC1 is commonly overexpressed in NSCLC cells, and pre-clinical studies have implicated MUC1 in driving epithelial-to-mesenchymal transition (EMT), immune evasion, and chemotherapy resistance [134–138]. Given its role in tumor progression and extracellular location, MUC1 represents a strong candidate TAA for therapeutic vaccination. Tecemotide (L-BLP25), a peptide encapsulated liposomal vaccine targeting MUC1, began clinical trials in NSCLC in the early 2000s. Early phase I results in 17 patients with advanced NSCLC demonstrated safety along with MUC1-specific CTL generation and immunogenicity *ex vivo* [139]. A subsequent phase II trial with advanced NSCLC showed a trend towards better median survival (4.4 months longer survival with L-BLP25 vaccination) [140]. The phase III START trial recruited 1239 patients with unresectable stage III NSCLC receiving vaccination with either concurrent or sequential chemoradiotherapy [141]. While sum OS showed no significant difference, patients receiving concurrent chemoradiotherapy displayed a moderate OS benefit (30.8 to 20.6 months, *p*-value = 0.016) [141]. The subsequent Phase III START2 administered Tecemotide with concurrent chemoradiotherapy; however, it was discontinued after a smaller Phase I/II trial in Japan saw no benefit of Tecemotide with concurrent chemoradiotherapy in terms of OS (NCT02049151) [142]. Other trials utilize Tecemotide in combination with other therapies following concurrent chemoradiotherapy, the most productive pairing to date being with the anti-vascular endothelial growth factor (VEGF) monoclonal antibody, Bevacizumab, where Phase II clinical trials have demonstrated tolerability and a trend towards better median PFS and OS [143].

### 5.2. Nucleic Acid and Viral Vaccines

#### Muc1

The early 2000s saw the generation of a modified, attenuated vaccinia Ankara strain (MVA), named TG4010, which encodes full-length MUC1 and human IL-2, as an adjuvant [144,145]. A Phase I trial for TG4010 began in a variety of solid tumors, including NSCLC, demonstrating vaccine tolerability in all 13 patients enrolled and disease stabilization in 4 of the 13 patients enrolled for 6–9 months [144,145]. A subsequent Phase II clinical trial in advanced NSCLC patients investigated TG4010 as a monotherapy (21 patients), or in combination with a first line chemotherapy regimen of cisplatin and vinorelbine (44 patients) [142,144]. Partial response was seen in 13 patients (29.5%) receiving combinatorial TG4010 with chemotherapy; however, only 2 patients receiving TG4010 as monotherapy experienced stable disease for more than 6 months [142]. An ensuing Phase IIb clinical trial compared TG4010 in combination with chemotherapy to chemotherapy alone and found greater 6-month PFS in the TG4010 plus chemotherapy group (43.2%, 32 of 74; 95% CI 33.4–53.5 *vs*. 35.1%, 26 of 74; 95% CI 25.9–45.3) [144,146].

These results culminated in the establishment of a Phase IIb/III clinical trial, TIME, initiated in 2012 to primarily assess the efficacy of TG4010 plus first-line chemotherapy among a larger cohort of advanced NSCLC patients with an activating EGFR mutation and >50% of tumor cells expressing MUC1 [147,148]. PFS in 111 patients receiving TG4010 and chemotherapy was 5.9 months (95% CI 5.4–6.7), while 111 patients receiving chemotherapy was 5.1 months (95% CI 4.2–5.9); *p*-value = 0.019 [147,148]. MUC1 specific T cells were predictive of better outcome, and epitope spreading to other TAAs was observed [147,148]. However, given the remarkable success of anti-PD1/PDL1 clinical trials around the same time, TG4010 trials were halted despite positive results.

### 5.3. Whole Tumor Cell Vaccines

#### 5.3.1. TGF-β

While TGF-β1 and TGF-β2 (known collectively as TGF-β) are not tumor antigens, they are major immunosuppressive molecules expressed by tumor cells and tumor-associated cells within the TME [88,106]. These molecules suppress both innate and adaptive anti-tumor immune responses, and the expression of TGFβ is correlated with worse prognosis and reduced response to immunotherapy in lung cancer [149]. Belagenpumatucel-L (Lucanix) is a vaccine generated from irradiated allogeneic NSCLC cells transfected with an antisense TGFβ2 gene plasmid to enhance adaptive anti-tumor immunity while downregulating TGFβ expression [150]. A phase II trial initiated in 2005 enrolled 75 stage II-IV NSCLC patients who received vaccinations once a month for 16 months [151]. The vaccine was well tolerated, while the 1 and 2 year estimated survival for high-dose groups were 68% and 52% respectively, while the low-dose group was 39% and 20% respectively [151]. These results formed the basis for the Phase III trial, STOP, enrolling 270 advanced NSCLC patients showing no progress after platinum-based chemotherapy to receive belagenpumatucel-L or placebo within 1 to 16 months following chemotherapy as a maintenance therapy [152]. The trial did not reach its survival endpoint, as there was no significant difference in OS (20.3 *vs*. 17.8 months, *p* = 0.594) or PFS (4.3 *vs*. 4.0 months, *p* = 0.947) between belagenpumatucel-L or placebo [152]. However, some positive indications of efficacy were found, as patients receiving vaccinations within 12 weeks of completing chemotherapy or prior radiotherapy demonstrated improved OS [152]. These findings suggest that vaccine approaches incorporating or combined with inhibition of TGFΒ signaling are promising.

#### 5.3.2. GVAX^®^ and Bystander GVAX^®^

GVAX^®^ is composed of autologous irradiated tumor cells that have been transfected with an adenoviral vector to secrete GM-CSF as an adjuvant [153,154]. A Phase I clinical trial in advanced NSCLC enrolled 35 patients in the trial, with successful GVAX^®^ vaccines generated for 34 of them. 18 of 25 assessable patients had DC, macrophage, granulocyte, and lymphocyte infiltration in tumors post-vaccination [153]. 3 of 6 patients had T cell and plasma cell infiltration with tumor necrosis within metastatic lesions resected post-vaccination [153]. In a separate phase I/II trial enrolling both early- and advanced-NSCLC patients, longer survival was seen only in patients secreting high levels of GM-CSF [155]. This suggests a threshold level of GM-CSF must be reached for vaccine efficacy.

A separate phase I clinical trial in metastatic NSCLC re-formulated the GVAX^®^ vaccine by adjuvanting autologous irradiated tumor cells with an allogeneic GM-CSF secreting cell line [156]. The vaccine was termed bystander GVAX^®^, and 49 patients were vaccinated out of 86 enrolled, displaying an increase in serum GM-CSF compared to the previous GVAX^®^ trial [156]. However, no patients achieved partial or complete response, with only 14% (*n* = 7) of patients maintaining stable disease for at least 12 weeks following initial vaccination [156]. No further exploration of GVAX^®^ has taken place in NSCLC largely due to the termination of two phase III trials using GVAX^®^ in prostate cancer (NCT00133224 and NCT00089856).

### 5.4. Summary

An evaluation of prior clinical trials employing therapeutic lung cancer vaccination reveals that, despite variations in antigen selection, vaccine formulation, and delivery platform, many vaccines have progressed through early-phase clinical trials and demonstrated safety coupled with a capacity to induce antigen-specific T cell populations in a portion of those enrolled (Table 1) [121,122,141,147,148,152]. The most promising results resulted from pairing vaccine-induced antigen-specific T cell generation with other established therapies, such as ICB, chemotherapy, or immunostimulatory adjuvants, underscoring the synergistic capacity and supportive role therapeutic cancer vaccines can play in tumor control [129,130,141,143,152–155]. Unfortunately, parlaying vaccine-induced antigen specific T cell responses into clinical benefits and improved disease prognosis has proven elusive. To date, all Phase III clinical trials have been unable to substantiate prior inclinations towards therapeutic efficacy suggested from earlier-phase studies.

There are a multitude of factors that are likely influencing this general trend of lung cancer vaccines showing safety and immunogenicity but ultimately lacking larger cohort-level efficacy in Phase II and III clinical trials [120–123,129,130,140,141,143,145–148,151,152,155]. When examining the similarities between the Phase III trials MAGRIT, START, START2, and TIME, only one tumor antigen was encoded in each vaccine (MAGEA3 for MAGRIT, and MUC1 for START, START2, and TIME) [121,122,141,147,148]. While MAGEA3 and MUC1 have been established as immunogenic tumor antigens capable of generating anti-tumor T cell responses, it is possible that encoding only one tumor antigen in a lung cancer vaccine formulation is not sufficient to broadly prolong survival [121,122,141,147,148]. Variance in patient expression of target antigens, loss of tumor antigen expression resulting from vaccine-induced selective pressure, or long-term T cell exhaustion could be contributing factors [96–101]. Additionally, assessments of memory T cell responses longitudinally following vaccination were not conducted, leaving the possibility open that vaccine-induced memory response generation is an absolute necessity for long-term vaccine efficacy to prolong survival.

The Phase III STOP trial on the other hand, combined TGFβ inhibition with a whole tumor cell vaccine approach encoding a wide breadth of tumor antigens [152]. Still, overall clinical efficacy was not observed, though exclusively looking at patients who recently received chemotherapy or radiotherapy before vaccination offered an increase in OS [152]. This result, in parallel with the MAGRIT, START, START2, and STOP trials, ultimately suggests that selection of a few, highly immunogenic tumor antigens in combination with adjuvants designed to suppress tumor cell activity or heighten T cell function may be a preferred strategy to ensure anti-tumor efficacy [122,141,147,148,152].

Ultimately, reviewing prior clinical trials in lung cancer vaccinology suggests that cancer vaccination can elicit anti-tumor immune responses. However, it may work best in combinatorial settings, pairing vaccine-induced T cell responses with other drugs more directly targeted towards tumor cell death (chemotherapy, radiotherapy, or ICB as examples). Additionally, incorporating robust assessments of T cell response kinetics pre-, during, and post-vaccination is critical for diagnosing potential issues with overall vaccine efficacy and memory response duration in future clinical trials that may preclude robust survival advantages across larger cohorts of patients.

The following section will now examine the current landscape of therapeutic lung cancer vaccination, focusing on recent Phase I trials and trials in preparation, with particular attention paid to the biological foundations guiding their design and the novelty in approach.

## 6. Current Therapeutic Vaccination Approaches in Lung Cancer

### 6.1. Peptide and Protein Vaccines

#### 6.1.1. Personalized Peptide-Based Vaccines

Beginning in the late 2010s, a significant focus has been placed on formulating individualized cancer vaccines tailored to the unique repertoire of TSAs and TAAs expressed on a patient-by-patient basis [157,158]. In 2020, results from a Phase Ib clinical trial were published combining PD-1 blockade with a personalized neoantigen based vaccine (NEO-PV-01) adjuvanted with the toll-like receptor 3 agonist, polyICLC, across a variety of solid tumors, including NSCLC [159]. Personalized vaccines were formulated by performing whole exome and RNA sequencing of patient-derived formalin-fixed tumor and matched normal cells from blood. Using bioinformatics, up to 20 antigenic epitopes were graded and selected primarily based on their predicted capacity to bind to human orthologs of MHC I molecules, Human Leukocyte Antigen-A and B (HLA-A and HLA-B) [159]. Of the NSCLC patients enrolled in the trial, an overall response rate (ORR) to vaccine + anti-PD-1 treatment of 39% (Confidence Interval (CI) = 17–64%) was achieved [159]. Additionally, identification of antigen-specific T cell responses against target antigens was observed in 47% of NSCLC patients [159].

A subsequent single-arm Phase Ib trial using NEO-PV-01 was initiated exclusively for non-squamous NSCLC patients, this time administered as a first-line therapy alongside a combinatorial immunochemotherapy regimen of pemetrexed, carboplatin, and anti-PD1 [160]. 38 patients were treated, and the regimen was determined to be safe and tolerable, with 43% of patients achieving a partial response (PR). However, determinations about vaccine efficacy alone are difficult given the multitude of treatment modalities administered and a single-arm approach [160]. Among PRs, there was an increased correlation of CD4+ and CD8+ T cells outside and within the tumor compared to non-PRs [160]. Following these positive results, future clinical trials should be designed to compare immunochemotherapy alone to immunochemotherapy + vaccination to determine the synergistic efficacy of personalized therapeutic vaccination with immunochemotherapy on lung tumor control.

A separate Phase I clinical trial is in preparation and looking to recruit advanced squamous NSCLC patients utilizing a personalized and dynamically adjusted neoantigen peptide vaccine (PANDA-VAC), including Poly-ICLC as an adjuvant and in combination with pembrolizumab (NCT04266730) [161]. Generation of PANDA-VAC will be through whole exome and single cell sequencing of patient archival tumor and matched normal samples, where 6 neoantigens will be selected for vaccine inclusion based on tumor specificity and HLA binding capability. Most intriguingly, after patients receive 5 prime doses and 2 boosters, observation of some sort of clinical response will initiate a subsequent round of tissue collection for possible vaccine adjustment and reformulation as novel neoantigens emerge during initial treatment. Given the collection of tumor and matched normal tissues for whole exome and RNA sequencing, pre- and post-treatment, crucial insights into the quality and nature of tumor neoantigen expression in response to cancer vaccination could provide the basis for other studies using a similarly dynamic personalized vaccine approach.

#### 6.1.2. Muc1

As a standalone TAA, MUC1 has garnered the most convincing clinical trial data suggesting its clinical efficacy [141,147,148]. Therefore, attempts to engineer vaccines encoding MUC1 are still under investigation. A Phase I/II study aiming to recruit 30 stage I–III NSCLC patients plans to use a MUC1 peptide vaccine adjuvanted with Poly-ICLC, which has shown safety and immunogenicity as a therapeutic vaccine for metastatic castrate resistant prostate cancer patients and a preventative vaccine for patients harboring advanced colonic adenomas (NCT01720836) [162,163]. In the phase II trial recruiting patients with recently diagnosed colonic adenomas that are not yet cancerous, a 38% absolute reduction in adenoma recurrence was observed in vaccinated patients who had an active MUC1-specific immune response at weeks 12 and 55 following initial vaccination compared to placebo (*p* = 0.08) [164]. Use of this vaccine for NSCLC will be more widely relevant as a therapeutic vaccination; however, this data provides rationale for possibly using this vaccine approach in a post-surgery setting to prevent or delay recurrence. There is also an investigation into the use of this vaccine as a prophylactic in smokers, who have elevated MUC1 levels [165,166].

### 6.2. Nucleic Acid Vaccines

#### 6.2.1. Multiple TAA-Encoded Vaccines

Previously mentioned clinical trials have established the capacity for single antigen encoded vaccines to generate antigen-specific immune responses, yet substantial clinical efficacy is largely lacking [122,141]. An important consideration for why single antigen encoded vaccines lack efficacy could be due to redundant compensatory mechanisms employed by tumor cells to promote immune evasion. As a result, many current clinical trials encode multiple antigens to target a multitude of pathways utilized by tumor cells for growth and survival. One such vaccine, CD105/Yb-1/SOX2/CDH3/MDM2-polyepitope plasmid DNA vaccine (STEMVAC) adjuvanted with GM-CSF, has demonstrated elevated antigen-specific T cell responses against a median of 4/5 vaccine-encoded antigens in a phase I clinical trial enrolling advanced breast cancer patients (NCT02157051) [167]. There is a planned phase II clinical trial exclusively recruiting stage IV NSCLC patients to test STEMVAC as a maintenance therapy in combination with pembrolizumab (NCT05242965) [167].

BNT116, a uridine RNA-based lipoplex vaccine encoding 6 TAA mRNAs (MAGEA3, CLDN6, KK-LC-1, PRAME, MAGEA4, and MAGEC1) began a first-in-human clinical trial in 2022 (LuCa-MERIT-1) recruiting 20 advanced NSCLC patients who have progressed on anti-PD1/PD-L1 and a platinum-based chemotherapy (NCT05142189) [168]. Vaccinations were performed in combination with docetaxel, where 7/20 patients had a partial response, and 10/20 achieved stable disease. The ORR was 35% (95% CI—15.4–59.2) and DCR was 85% (95% CI—62.1–96.8) [168]. Antigen-specific T cell responses were observed via ELISpot and cytokine analysis [168]. A phase II trial, EMPOWERVAX Lung 1, is currently recruiting advanced NSCLC patients with greater than or equal to 50% PD-L1 expression on tumor cells to receive BNT116 in combination with the anti-PD1 mAb, cemiplimab, or cempilimab alone (NCT05557591) [169]. Together, these initial trials have established that multiple antigen-specific T cell populations can be generated with one vaccine. Further expansion into larger clinical trials to establish clinical benefits of multiple TAA-encoded vaccines will require careful recruitment criteria and multiple-arm approaches to implement vaccination with other therapies.

#### 6.2.2. Personalized Neoantigens

KEYNOTE-603, a phase I trial, evaluated safety and immunogenicity of mRNA-4157 (V940) combined with pembrolizumab in 4 resected NSCLC and 12 resected cutaneous melanoma patients [170]. V940 encodes up to 34 tumor-specific neoantigens, selected via whole-exome sequencing and computational modeling of patient tumor and healthy tissue [170]. Patients received treatment following surgical resection with the intention of preventing or delaying tumor recurrence [170]. The ensuing phase IIb trial, KEYNOTE-942, enrolled completely resected high-risk melanoma patients to receive V940 with pembolizumab (*n* = 157) or pembrolizumab alone (*n* = 50) [171]. V940 vaccination showed an improved 18-month recurrence-free survival compared to pembrolizumab alone (79% versus 62%) [171]. These results provided a compelling rationale motivating an upcoming phase III trial, INTerpath-002, exclusively recruiting NSCLC patients to compare pembrolizumab with or without V940 vaccination following surgical resection, with the primary outcome measuring disease-free survival after resection (NCT06077760) [172].

Autogene cevumeran is a uridine messenger RNA lipoplex-based vaccine encoding up to 20 personalized neoantigens determined through whole exome and RNA sequencing, followed by in silico prediction analysis of patient tumor and blood samples collected during surgical resection [173–175]. Initial Phase I trials were performed in pancreatic cancer patients as an adjuvant following atezolizumab (anti-PD-L1) and preceding a four-drug chemotherapy regimen (mFOLFIRINOX), 8 out of 16 enrolled patients had robust generation of neoantigen-specific T cells [173,174]. Of the 8 responders, there was a significantly longer median RFS (not reached) versus non-responders (13.4 months, *p* = 0.007) [173,174]. These promising results suggest that personalized cancer vaccination could play a key supportive role following surgical resection, when disease burden is minimal, to prevent recurrence. Based on this positive data, a subsequent Phase I clinical trial enrolling advanced solid tumor patients, including 28 NSCLC patients, was vaccinated with autogene cevumeran with or without atezolizumab [175]. The study was not designed to evaluate clinical efficacy; however, generation of durable CD4+ and CD8+ T cell responses was observed in roughly equivalent frequencies between autogene cevumeran alone or with anti-PD-L1 (11/15, 71% and 53/75, 71%, respectively) [175]. Subsequent Phase II studies are currently ongoing or recruiting in advanced melanoma, pancreatic cancer, and colorectal cancer, with the demonstrated data providing rationale for studies in NSCLC, as well (NCT03815058, NCT05968326, and NCT04486378).

Though direct studies of personalized mRNA vaccines in lung cancer are limited thus far, emerging evidence across all cancer types has demonstrated clear feasibility and immunogenicity using such an approach. Given the speed and feasibility with which surgically resected tumor tissue can be processed and sequenced for generating mRNA encoding patient tumor-antigens, fast-tracking phase III trials in lung cancer are both reasonable and critical to determine survival benefits. It is likely that patients with a larger TMB, or with higher quality antigens to select from will have a greater benefit, so additional measures to systematically evaluate the magnitude and likelihood of patient benefit is another important step for utilizing personalized vaccine technology.

### 6.3. KRAS Neoantigen

In 2019, a Phase I/II clinical trial recruiting advanced solid tumor patients, including 6 NSCLC patients, combined ICB with a heterologous vaccination strategy combining chimp adenovirus (ChAd68) and self-replicating RNA (srRNA) coding for 20 shared neoantigens derived from common KRAS and TRP53 mutations [176]. The vaccine was named SLATEv1, and the Phase I arm of the trial evaluated safety, immunogenicity and clinical response [176]. The vaccine was well tolerated, though disease progression was detected in 15/19 patients [176]. Median PFS was 1.9, while median OS was 7.9 months, providing some indications of clinical responsiveness [176].

All patients in the study harbored KRAS mutations; however, generation of KRAS neoantigen-specific T cells detectable by *ex vivo* IFN-γ ELISpot of PBMCs was only detected in 5/16 analyzed patients, TRP53 neoantigen expression was detected in 10/12 analyzed patients [176]. This observation led to the intriguing idea that HLA-matched neoantigens influence therapeutic response, with broader implications for optimizing future cancer vaccine designs [176]. Further pre-clinical assessments revealed that vaccinating mice with a KRAS-focused neoantigen cassette resulted in much stronger generation of KRAS neoantigen specific T cells than SLATEv1 vaccination, which was lost upon adding HLA matched or unmatched TRP53 neoantigens back into the cassette [176]. This suggests that regardless of tumor antigen expression, incorporation of multiple antigens in vaccine design can promote competition and possibly dampen anti-tumor immunity [176]. Additionally, inclusion of 4 copies of each KRAS neoantigen into the KRAS-focused cassette further elevated the KRAS neoantigen-specific T cell response, suggesting antigen density can amplify the magnitude of anti-tumor T cell responses [176]. Based on these data, SLATEv1 has since been redeveloped only to contain the 4 most highly prevalent KRAS neoantigens, repeated 4 times each, and was renamed SLATE-KRAS. A phase II clinical trial is currently recruiting patients harboring advanced solid tumors to combine SLATE-KRAS vaccination with KRAS-specific autologous T cell therapy (NCT06253520).

### 6.4. Dendritic Cell Vaccines

While mRNA-based vaccine approaches have become the premier method of antigen delivery following the remarkable successes of the COVID-19 vaccine, DC vaccines still require further evaluation due to their efficiency at cross-presenting exogenous antigens, enabling more efficient and broad T cell activation [64,66]. This process, coupled with DC-derived proinflammatory cytokine secretion that functions as an adjuvant, can produce robust type I effector T cell responses [177,178]. With growing interest in personalized cancer vaccines encoding patient-specific tumor antigens, the potential role of DC vaccines in this context requires further evaluation.

A phase I clinical trial recruited 10 NSCLC patients without oncogenic driver mutations or gene rearrangements (EGFR/ALK/ROS1/RET) eligible for surgical resection [179]. Following surgery, tumor tissue and blood were collected from patients for sequencing and in silico neoantigen prediction and selection [179]. Neoantigen-encoding mRNA-LNPs were then produced for the generation of patient-derived, mRNA-loaded monocyte-derived type 1-polarized DCs, constituting the MIDRIXNEO vaccine [179,180]. MIDRIXNEO was successfully generated for 6/10 patients with a median time of 198 days between surgery and vaccine formulation (range, 152–246 days) [179]. The vaccine was well tolerated, and vaccine-induced T cell responses were observed in 5/6 patients for 14/33 (42%) neoantigens, as assessed by *ex vivo* peptide stimulation of patient PBMCs [179]^.^ Future clinical trials with a larger cohort of patients paired with orthogonal approaches to understand immune dynamics following vaccination will be critical to establish the clinical benefits of MIDRIXNEO vaccination and possible unique immunological advantages to personalized DC-based cancer vaccination.

### 6.5. Summary

Building upon past failures and successes, the current iteration of therapeutic lung cancer vaccines is collectively addressing and providing answers to key questions that may help to yield clinical benefits ultimately (Table 2). Namely, the independent usage of rapid high-throughput tumor tissue RNA and DNA sequencing combined with in silico predictive modeling to generate personalized peptide (NEO-PV-01 and PANDA-VAC), mRNA (V940), and DC (MIDRIXNEO) vaccine formulations has shown the feasibility and practicality of such an approach [160,161,170,179]. KEYNOTE-603 and -942 demonstrated the potential for personalized vaccines to be developed and deployed in a post-surgery, minimal disease state with the intention to prevent or delay recurrence in a role that may be optimal for therapeutic vaccines [170,171]. The succeeding phase III INTerpath-002 trial will determine the clinical potential of this approach in an expanded cohort of patients. It is critical for future trials using personalized vaccines to perform robust analyses of immunological responses pre-, during, and post-treatment to determine the kinetic response to treatment and durability to promote long lasting anti-tumor immunity. Further in depth phenotypic and clonotypic analyses using high throughput sequencing and flow cytometric techniques are also imperative to understand molecular and cellular determinants of response, such as TMB, antigen quality, TCR specificity, T cell effector function, and TME composition that may provide insights into patient tumor and immune characteristics to optimize or indicate the likelihood of successful treatment. Careful considerations towards combinatorial therapeutic agents are also key, as ICB and chemotherapy represent just a couple of viable agents that could improve patient outcomes if strategically combined with vaccination.

Beyond personalized vaccines, these types of analyses and insights also apply to fixed antigen vaccines broadly applicable to wider subsets of patients. Benefits to this approach include immediate ‘off-the-shelf’ usage for patients, well-established and characterized immune reactivity, coupled with the ease of storage for deployment in community care clinics. The upcoming STEM-VAC, 1503TiP, and SLATE-KRAS clinical trials hold particular interest as multiple, high quality, tumor antigens were included in each formulation, and an impressive trial design will allow the benefits of vaccination alone to be derived.

Despite notable potential for the current iteration of therapeutic lung cancer vaccines, it remains to be seen whether these new approaches will yield clinical benefit to ultimately prolong patient survival and quality of life, which has proven elusive thus far. Additionally, determining optimal vaccine delivery platforms, adjuvants, and combinatorial therapies will not be directly addressed with the current crop of clinical explorations. The next section will now look towards active areas of pre-clinical study that will aid future vaccine design.

## 7. Future Therapeutic Vaccination Promises in Lung Cancer

### 7.1. Artificial Intelligence (AI)

The increasing promise for personalized vaccines underscores a crucial need for more efficient and accurate computational models and pipelines that synthesize patient tumor sequencing data for optimized vaccine formulation. Recent breakthroughs in AI have transformed our ability to derive biological insights from large-scale patient tumor WES and scRNA-seq data, playing a key role in the present and future of cancer vaccine design [2–6,181,182]. While there are concerns regarding the use of AI technology in place of human ingenuity and work, a reasonable application for AI regards its use in high-dimensional pattern recognition and data synthesis [183]. In this fashion, AI could be pivotal in synthesizing prior clinical trial datasets, predicting patient benefits from vaccination, refining antigen and epitope selection based on tumor expression and HLA status, enhancing synergies with other treatment modalities, and identifying effective adjuvants, ultimately strengthening the effectiveness of cancer immunotherapy and vaccinology.

Natural language processing (NLP) is being implemented to interpret better and standardize unstructured data resulting from electronic health record documentation of immune-related adverse events (irAEs) to ICB [184,185]. Simultaneously, machine learning (ML) approaches are being used to synthesize human and mouse datasets to predict the patient populations most likely to benefit from ICB [186–188]. By leveraging data from prior clinical trials, NLP could help identify optimal tumor antigens and adjuvants, providing valuable insights for future treatment strategies and enhancing therapeutic success [189–191].

Regarding optimal antigen formulations, ML approaches have been developed to interpret patient tumor and immune profiles, which may offer more accurate predictions on optimal antigen selections [169,192,193]. Multiple ML models have been developed and trained using highly controlled murine models to predict antigen quality based on epitope immunogenicity, HLA binding affinity, and biochemical peptide: HLA properties [194–201]. Currently, there is no gold standard pipeline for assimilating multiple ML models analyzing different parameters to inform vaccine design. Such an approach would require human intervention at each step of data assimilation to ensure accurate and relevant predictions. Deep learning (DL) could help address this challenge by offering a multi-modal AI language model in which all vaccine-relevant information is assimilated and trained together, without the requirement for human intervention [190,202–204].

The future of AI to aid clinical trials and vaccine design, particularly for personalized medicine, holds incredible promise. To fully realize its potential, key challenges must be addressed, including standardizing tissue collection and model training procedures, developing integrated pipelines and DL models that incorporate multiple AI algorithms into vaccine prediction models, and providing guidelines and regulations for the application of AI in cancer care. Advancing these elements and continuing to innovate and improve AI models is an essential step for fueling the next generation of cancer vaccines.

### 7.2. Lipid Nanoparticles

Lipid-based encapsulation systems, most notably lipid nanoparticles (LNPs), have become increasingly popular for cancer vaccine designs due to their adjuvant activity, durability and retention in tissues paired with customizability and ease in manufacturing [62,205–208]. LNPs build on previous lipid encapsulation systems by incorporating ionizable lipids that facilitate endosomal escape and cargo release after cellular uptake in a pH-dependent manner [62,209–212]. Another big reason for the focus towards LNP-based cancer vaccines is the enhanced permeability and retention (EPR) effect, a phenomenon by which solid tumor vasculatures promote accumulation and retention of nanoparticles and other nano-scale drugs in tumor tissues [213–216]. Improving the design of carrier molecules to promote increased antigen presentation, immune stimulation, and retention in lymphoid or diseased tissues is an active area of investigation to enhance vaccine efficacy [170,173–175,217].

Two methods of study for improving LNP delivery surround passive or active targeting of APCs [218]. Passive targeting of LNPs to APCs requires increasing particle size to preferentially target phagocytic uptake by APCs for subsequent antigen presentation [213,218–222]. Polyethylene glycol (PEG) lipids, a common component of LNPs that help reduce innate immune clearance and prevent particle aggregation, can be lengthened or shortened to alter circulation and cargo release times [223,224]. DC specific uptake has also been demonstrated by adjusting the net charge of mRNA-lipoplex nanoparticles by adjusting the ratio of negatively charged mRNA to positively charged cationic lipid components [225]. BNT111, a melanoma mRNA-lipoplex vaccine targeting four TAAs (NY-ESO-1, MAGE-A3, tyrosinase, and TPTE), has already demonstrated safety and immunogenicity in the phase I clinical trial, Lipo-MERIT for advanced melanoma patients [226].

However, there is a fine balance when generating LNPs to target APCs passively. PEGylated LNPs have been observed to promote the accelerated blood clearance effect, an antibody mediated phenomenon that promotes rapid clearance of LNPs after initial vaccination [227–229]. Passive targeting approaches also currently lack tissue specificity and could lead to enhanced accumulation in the liver, given the density of macrophages within hepatic tissues. This problem already exists with current LNP delivery methods [230–233]. Measuring the impact of antibody-mediated clearance and tracking the distribution of nanoparticle vaccines will be a key consideration in pre-clinical models and early phase clinical trials to improve therapeutic efficacy.

Active targeting of LNPs to APCs requires changes to lipid formulations or protein conjugations to facilitate receptor-binding activity and proximity-based interactions [205,217,218,234]. Mannosylation of ionizable lipids have been demonstrated to increase DC uptake and tumor control in murine models by promoting cellular uptake through the endocytic mannose receptor (CD206), predominantly expressed by APCs [235–239]. PEG lipids can be modified to facilitate specific targeting by acting as a linker to conjugate cell-targeting antibodies [225,240–242]. Conjugation of LNPs with antibodies has been demonstrated in vaccines encapsulating short interfering RNA (siRNA) to target APCs, including for CD206, however, approaches for mRNA- or peptide-based vaccines have yet to be developed [242,243]. Pertinent to lung cancer, siRNA-LNPs conjugated to F480 antibodies have been used intranasally in mouse models to deliver cargo selectively targeting lung macrophages [244]. Selective organ targeting (SORT) LNPs have also been developed to preferentially target lung tissue in mice by changing the surface chemistry of the outer LNP shell [245–248]. SORT LNPs were demonstrated in mouse models to gene edit >70% of lung stem cells and >80% of lung epithelial cells for around 2 years [249,250]. Furthermore, SORT-LNPs encoding Cas9 mRNA were able to correct cystic fibrosis *CFTR* driver mutations in patient derived cells, organoid models, and mouse models [249,250]. Now SORT-LNPs (RCT2100) are being used in a phase I clinical trial for cystic fibrosis patients, delivered via inhalation (NCT06237335). It is currently unknown whether the lung TME may interrupt or limit lung tissue targeting LNPs, however, the promise these approaches hold is immense and requires detailed investigation. One logical advantage of this targeted approach in lung cancer is to establish local resident memory T (T_RM_) populations that can extend the duration of vaccine-induced anti-tumor immune responses locally. However, it remains to be seen whether targeted LNP approaches are better at inducing T_RM_ responses.

### 7.3. RNA Modifications

Advances to antigen-encoding RNA have also been developed to build upon traditional mRNA-based approaches to increase antigen load, stability, and immunogenicity [205,206,217,251]. Self-replicating RNA (srRNA) vaccines, as used in the SLATEv1 clinical trial, are an emerging technology constructed using antigenic RNAs formulated as a positive-strand RNA virus containing replicase that form viral replicon particles (VRPs) upon supplementation of structural proteins [176,251–255]. These VRPs then undergo replication upon infecting a target cell that drives vaccine-encoded tumor antigen generation in situ in a more sustained manner than mRNA, which has a relatively short half-life [176,252–254,256]. Commonly, srRNA vaccines are used after prior administration of a non-replicating vector vaccine as a booster to elevate pre-existing adaptive immunity established through memory T cell generation [176,254,257–260].

Phase I clinical trials using srRNA vaccines encoding TAAs have been performed in a variety of cancers, including lung, colorectal, breast, and esophageal cancer, demonstrating safety and immunogenicity [260–263]. To establish the clinical response to srRNA vaccination, Phase II trials are ongoing in NSCLC (SLATE-KRAS) and metastatic colorectal cancer (GRANITE) in combination with ICB (NCT06253520, NCT05141721) [264]. Designing the trials to interrogate the efficacy of srRNA vaccination alone will be key to establishing the therapeutic benefit of such an approach, while synergies with ICB are also pivotal to optimize anti-tumor immunity and establishing synergistic therapies. It is also of great interest to establish how effective srRNA vaccines are at boosting pre-existing memory T cell responses or expanding new memory cells. Assessing memory T cell responses and overall T cell response kinetics pre- and post- srRNA administration will be pivotal in assessing the capacity for self-replicating vaccines to induce durable memory responses.

Another emerging RNA-based vaccine technology is circular RNA (circRNA), single-stranded RNAs that form a closed-loop structure via covalent linkage between the 5′ and 3′ ends of the molecule [265–267]. CircRNAs have much greater stability than linear RNA approaches and are more immune quiescent, making them an easier and more durable method of RNA-based vaccination [256,266–270]. LNPs have been the primary encapsulation method studied for circRNA delivery; however, naked delivery of circRNA has also been reported to show efficacy in mice given the inoculation site contains an appropriate density of APCs, raising the possibility for intratumoral injections in settings where patients have a high density of APCs within their TME [271,272].

Despite no clinical trial data demonstrating the efficacy of circRNA-based vaccines, pre-clilnical studies in mice and non-human primates have shown promising results across a variety of disease models, including cancer [265–268,271–273]. Direct comparisons between circRNA and mRNA vaccines in pre-clinical models have shown the improved capacity of circRNA to generate better antibody responses and cross-reactivity against SARS-CoV-2 variants than current modified mRNA methods [265]. Studies using tumor models are sparser, however, circRNA expressing the model tumor antigen, ovalbumin (OVA), was used in mice bearing orthotopic melanoma (MC38 or B16) [274,275]. Vaccination was able to limit both primary tumor growth, but also the metastatic spread of B16 tumor cells to the lungs [274,275]. To translate this emerging technology for human applications, further improvements in manufacturing and encapsulation methods are required before advancing to clinical trials.

Given the strengths and weaknesses of circRNA and srRNA for vaccination, it is conceivable that heterologous approaches using a circRNA-prime vaccine to initiate anti-tumor immunity are superior, given the improved durability, while boost approaches using srRNAs could be ideal to increase the overall antigen load *in situ*. However, while srRNA approaches are actively being utilized in clinical trials, circRNA-based vaccines have not yet reached the phase of clinical trial, likely due to issues with larger-scale manufacturing and safety validation. Regardless, improving RNA-based vaccine delivery platforms is essential given their growing utilization, relative ease in manufacturing, and adaptability for personalized medicine. The unique advantages of srRNA and circRNA approaches represent the logical progression for RNA-based vaccine platforms and the next generation of lung cancer vaccines.

### 7.4. Mucosal Vaccination

An intriguing aspect of lung cancer vaccinology centers around the selective mucosal barrier separating the lungs from the respiratory lumen [276–278]. Physiologically, the respiratory mucosa is essential for pulmonary function, mediating airflow through the respiratory tract into the alveoli to facilitate gas exchange [279,280]. Immunologically, the respiratory mucosa plays a critical role in continuously interpreting external immunogenic cues to properly balance local tolerogenic and inflammation [281–286]. This immune balance is achieved in part through the generation of iBALT, which facilitates antigen presentation and B and T cell activation locally in the lungs to respond rapidly to insults [287–292]. Contributions from both iBALT and systemic lymphoid tissues following pathogenic insult to the lungs result in the generation of resident memory B cell (B_RM_) and T_RM_ populations that enable rapid and robust expansion of antigen-specific T cell populations in previously insulted lung tissue following antigen re-encounter [290,293–300]. T_RM_s and iBALT structures have been observed in all stages of NSCLC and are associated with better prognosis [42,44]. Thus, the immune response to lung cancer is the sum of both mucosal and systemic inputs. However, the cellular and molecular cues that drive iBALT formation, as well as the capacity for cancer vaccines to induce or enhance iBALT structure functionalities, are currently unknown.

A key, yet unresolved question is whether direct therapeutic vaccination of the lungs via inhalation or intranasal vaccination represents a more optimal route for inducing anti-tumor immunity against lung cancer than contemporary systemic routes. Mouse studies have demonstrated that intranasal vaccination elicits both cellular and humoral immune responses with greater magnitude than systemic vaccination, conferring enhanced protection from infection and reinfection [284,299,301]. Notably, a systemic-prime followed by mucosal-boost vaccination strategy significantly amplifies lung-specific adaptive immunity, offering even greater protection [284,299,301]. These findings contributed to the approval of an intranasal COVID-19 vaccine using an adenoviral vector in China and India, which demonstrated safety and recapitulated the improved protective effects observed in mice [302–304]. Currently, a phase I trial is evaluating this vaccine formulation in the U.S. via both intranasal and inhalation routes (NCT06441968). However, for mucosal vaccine approaches to reach early-phase clinical trials in lung cancer, additional pre-clinical studies demonstrating improved survival and overall anti-tumor immunity to contemporary systemic boosting approaches are crucial.

From an immunological standpoint, a systemic prime followed by a mucosal boost is a logical approach for lung cancer. Initial systemic priming would seed precursors of T_RM_ and B_RM_ cells in the lung and associated tissues, enhancing the persistence and magnitude of subsequent mucosal vaccination [283,299,300,305]. One such benefit is the local enhancement of respiratory T_RM_ and B_RM_ responses through pre-existing CD4+ T cell help [300,305,306]. This effect could be in part driven by the generation of vaccine-induced iBALT or iBALT-like structures to drive local antigen presentation and T cell activation *in situ*. However, there is no such evidence to date yet. In preclinical models, prophylactic mucosal vaccination encoding tumor antigens has been shown to establish T_RM_ populations in the lungs that prevent lung tumor formation following tumor challenge [307,308]. Therapeutic systemic-prime followed by mucosal-boost vaccination additionally has been shown to delay tumor growth when administered following intravenous tumor cell challenge [309]. However, many questions remain unaddressed that need answers before clinical implementation. One major consideration is the ability of vaccine particles to efficiently traverse the respiratory mucosa for antigen delivery, followed by effective effector B and T cell migration into the TME. Unlike systemic vaccination, mucosal vaccination must penetrate a highly viscous mucosal layer rich in mucins, which enzymatically degrade foreign materials such as mRNA, LNPs, and viruses [310,311]. To overcome these obstacles, novel LNP formulations and adjuvants with enhanced resistance to mucosal degradation have been developed, and circRNA has also shown increased durability in the respiratory mucosa compared to linear mRNA [312–314].

Upon successful vaccine penetrance into the lungs, additional challenges arise from the immunosuppressive nature of the TME, which can limit antigen presentation efficiency, immune cell infiltration, memory response generation, and CTL activity [25,81,82,85]. Addressing these barriers will require detailed patient TME analyses, strategic adjuvant selection and combinatorial approaches aimed at stimulating inflammatory cytokine production and T cell effector activity. Moreover, systemic blood sampling may be insufficient for assessing mucosal vaccine responses, as immune activity is predominantly localized within the lung parenchyma [315–317]. Since systemic blood sampling may not fully capture mucosal vaccine responses, alternative strategies such as minimally invasive respiratory sampling, lung imaging integrated with immune profiling, or analysis of tumor draining lymph nodes may provide more informative assessments of vaccine efficacy. However, it remains unclear whether mucosal vaccination directly stimulates tumor-draining lymph nodes, highlighting the need for future investigation.

**Figure 2.**
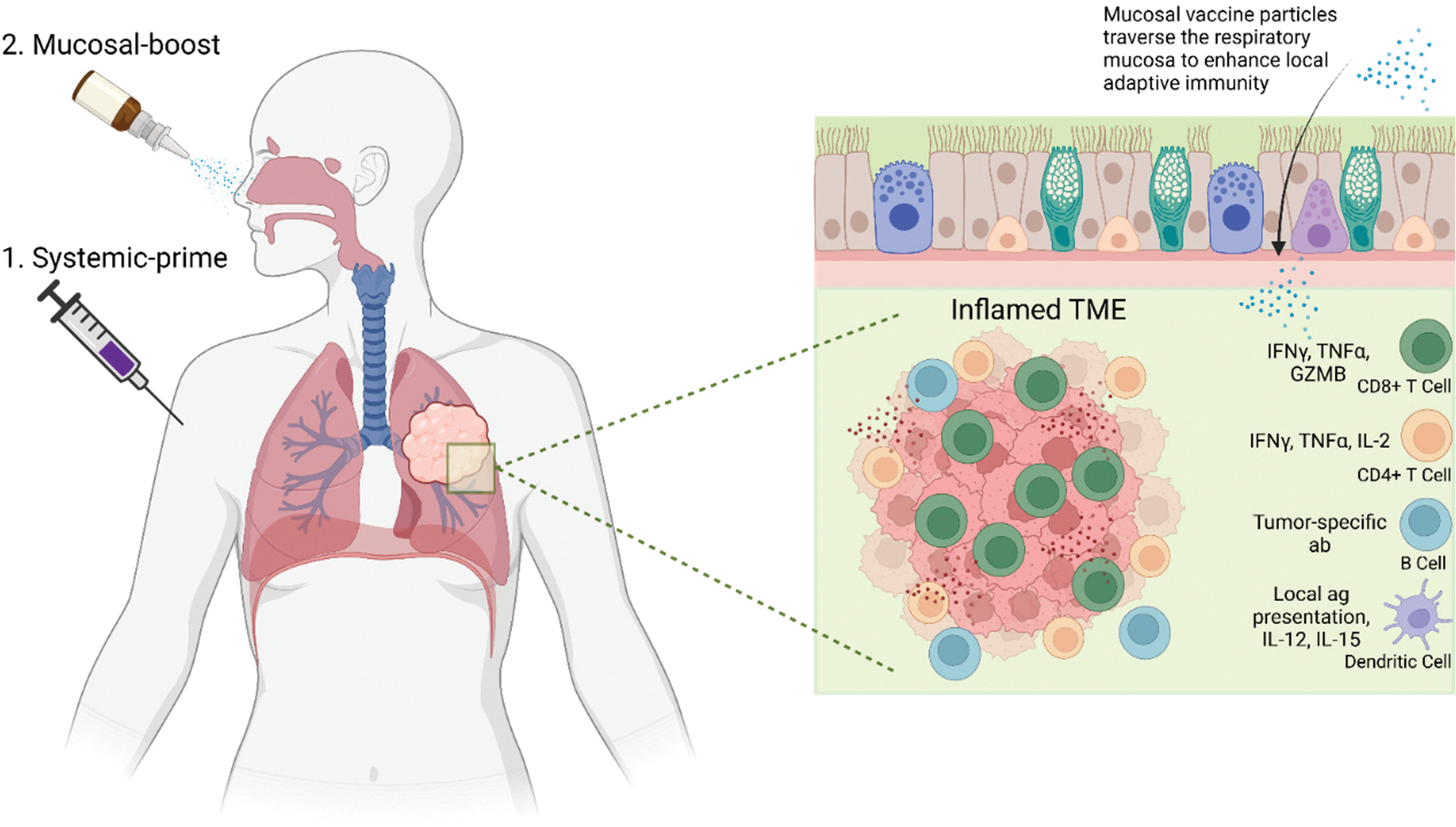
Mucosal Vaccination Diagram. Systemic-prime plus mucosal-boost vaccination strategy could selectively enhance local lung anti-tumor immunity. By directly promoting vaccine particle uptake in the lung epithelium, local antigen presentation by dendritic cells can lead to faster and better adapted anti-tumor CD4+ and CD8+ T cell responses. This better adaptive anti-tumor immune response could lead to a more inflamed lung TME, with greater immune infiltration and pro-inflammatory cytokine production resulting in more tumor cell killing. TME: Tumor microenvironment; IFNγ: Interferon-γ; GZMB: Granzyme B; TNFα: Tumor Necrosis Factor-α; IL: Interleukin; ab: antibody.

Given the feasibility of direct lung vaccination, there is a unique opportunity present to explore how local vaccine stimulation of APCs and resident memory populations impacts anti-tumor immunity (Figure 2). Additionally, it remains to be determined whether this approach offers superior therapeutic efficacy compared to systemic vaccination alone. The enhanced effectiveness of mucosal vaccination following prior systemic priming suggests that heterologous vaccination strategies could optimize both systemic and mucosal anti-tumor immunity. One potential approach could involve the use of srRNA to induce a robust systemic response with high initial antigen load, followed by circRNA for mucosal delivery, leveraging its enhanced durability in the respiratory tract. Further pre-clinical studies are necessary to refine vaccine formulations, determine optimal dosing and timing, and elucidate key cellular and molecular mechanisms underlying efficacy while identifying potential pitfalls.

## 8. Discussion

Despite many attempts to develop therapeutic vaccines for lung cancer, each using novel formulations, antigens, delivery methods, adjuvants, combinatorial agents, and sequencing regimens, there is no clinically approved vaccine nor established widespread clinical benefit. However, it would be unwise to suggest that progress has not been made, with past failures providing key insights and rationale for current-day approaches that are showing increasingly promising results, coupled with emerging technologies, such as personalized medicine, that will one day fuel the next generation of clinical trials.

Past therapeutic lung cancer vaccination attempts clearly demonstrated the capacity for vaccine formulations to safely induce and amplify tumor antigen specific T and B cell responses—all detectable through patient blood sampling. These responses provided indications of therapeutic benefits for many patients, particularly in combination with other treatments like ICB or chemotherapy, leading to several Phase III clinical trials, including MAGRIT, START, START2, TIME, and STOP [121,122,141,147,152]. Unfortunately, all trials were terminated after being deemed inferior to ICB or due to a lack of significant cohort-level clinical efficacy compared to placebo or a combinatorial agent alone.

A common theme for 4 of the Phase III trials (MAGRIT, START, START2, and TIME) is that only one highly immunogenic TAA was selected for each formulation, placing immense pressure on it to remain highly expressed by tumor cells and presentable on MHC molecules [121,122,141,147]. There has been a shift in current day approaches towards either personalized vaccine formulations encoding multiple neoantigens, or off-the-shelf formulations consisting of multiple well-defined tumor antigens to offer greater breadth to vaccine-induced anti-tumor immune responses. However, it has also been observed that immunodominance hierarchies can emerge when vaccinating with multiple unique tumor antigens that limit therapeutic effect [176]. Recent breakthroughs in AI technology may help to optimize future vaccine design given its ability to uniformly decipher and integrate complex clinical trial datasets and provide accurate predictions about antigen selection, vaccine formulation, and treatment regimens [182,190].

Additionally, 3 of the trials (MAGRIT, START, START2) used peptide-based formulations that provide further immune constriction by preprocessing the TAA for immediate presentation on MHC molecules [121,122,141]. While peptide-based vaccine formulations are still widely used, there has been a noticeable shift towards RNA-based vaccination strategies that facilitate epitope selection in situ by the patient’s own immune system [67,68].

An open question remains regarding whether constraining T cell responses to a few, highly immunogenic antigen epitopes via peptide-vaccination is preferable to vaccination strategies that rely on cross-presentation to generate a more diverse antigenic repertoire, such as RNAs. The overall efficacy for each formulation likely hinges on three rate limiting steps: (i) the tumor-intrinsic capacity to generate a sufficient range and scope of TAAs and TSAs presentable to both CD4+ and CD8+ T cells; (ii) the tumor-extrinsic aptitude for DCs to cross-present tumor antigens to prime sufficient anti-tumor T cell migration and effector responses leading to (iii) the total number of antigen-specific T cells capable of productive anti-tumor actions within the TME. A direct clinical trial comparing peptide to RNA vaccines utilizing personalized neoantigens or encoding the same TAAs may also be warranted to answer this question. Mileage will likely vary by individual patient tumors, making the appropriate delivery mechanism an important consideration in personalized cancer therapy alongside antigen selection.

Recent innovations in LNP-based delivery platforms and mucosal vaccines could make direct vaccination of the lungs a viable future approach. Pre-clinical studies have led to the development of lung-targeting LNPs that have the potential to revolutionize lung cancer therapy [245–248]. Likewise, mucosal vaccination has proven an effective approach to prevent viral infections, particularly in combination with prior systemic vaccination, however, its capacity to serve as a therapeutic lung cancer vaccine is currently unknown [284,299]. Despite pre-clinical demonstrations for mucosal vaccination to generate T_RM_ populations capable of tumor killing activity, the studies were done in models utilizing intravenous tumor cell challenge that do not fully recapitulate typical lung tumor formation or metastatic establishment [307–309]. However, it does raise an intriguing point as to whether local vaccination can offer greater protection from metastasis to and from the lung. For all lung targeting vaccine approaches, further questions remain regarding their capacity to induce anti-tumor immunity *de novo* in lung tissue or in nearby tdLNs, as well as the extent and depth to which APCs are capable of presenting vaccine delivered antigens. It is also important to compare these direct approaches to current systemic methods to ensure therapeutic improvement on current approaches. Exploration of new or redeveloped adjuvant formulations may also be required, given the balance between optimizing immunogenicity and ensuring stable lung function.

When taking in all the immunological mechanisms underlying therapeutic cancer vaccine efficacy, how they have fared in clinical trials thus far, what key advancements current approaches are taking, and how pre-clinical studies are innovating for the next generation of vaccines, the future role for therapeutic cancer vaccination and steps required to establish clinical approval begin to take shape. The presented studies and literature make a strong case for therapeutic lung cancer vaccination to play a supportive role in minimal disease states, such as post-surgery or in early-stage disease, with the intent to prevent or delay recurrence. When combining vaccination with chemotherapies or ICB agents that promote direct tumor regression, there is a clear path towards improved survival and quality of life, even if curative intent is not ultimately achieved. Vaccination in combination with ICB presents a strong immunological synergy, given vaccination can induce increased populations of T and B cells in circulation and within the TME, where ICB can then elevate their functionality and tumor killing capacity [16–18,173–176].

To achieve this and ultimately strive for curative intent in a manner that benefits the most patients possible, further integration of personalization into all aspects of vaccine design, formulation, delivery, and overall treatment strategy is the optimal path (Table 3). However, access to such care may not be available for all patients at least initially, and continued exploration for vaccine formulations encoding diverse high-quality, widely expressed TAAs that can be administered in local care facilities is also crucial. Continued pre-clinical innovation, open communication about successes and failures of ongoing clinical trials, quicker escalation into phase III/IV trials following success in other cancer types and carefully designed clinical trials evaluating both patient clinical and immunological responses are key to establishing cancer vaccination as a viable therapeutic strategy.

## 9. Conclusions

Lung cancer is a highly heterogeneous disease, arising from diverse cell types and evolving through complex perturbations in essential growth and proliferative pathways [2–6,9]. As a result, the antigenic repertoire expressed from an individual lung tumor varies widely, and in combination with an immunosuppressive TME, makes immunotherapeutic manipulation difficult to maximize for each patient [75,81,82]. This has been especially evident in therapeutic vaccination efforts, as no clinical trial to date has progressed beyond Phase III, despite consistently demonstrating safety and immunogenicity. However, the latest generation of vaccines entering clinical trials offers renewed optimism by leveraging novel innovations, such as personalized antigen selection algorithms, mRNA-LNP formulations, and adjuvants that could only have been informed through the failures of the past [159,161,168,170,173–175]. While progress has been slower than initially hoped, the landscape of therapeutic lung cancer vaccination is evolving rapidly. The integration of emerging technologies, such as AI-driven antigen selection, LNPs, srRNA, circRNA, mucosal vaccines, and novel combination strategies, will undoubtedly define the level of success for future trials [182,190,217,254,284]. With continued innovation and refinement, vaccines have the potential to expand the immunotherapeutic arsenal against lung cancer, ultimately improving patient outcomes.

## Figures and Tables

**Table 1.** Past clinical attempts in lung cancer vaccinology.

Clinical Trial Name	Vaccine Name	Phase	Formulation	Antigen	Cancer Types	Enrollment	Status	ClinicalTrials.gov ID
MAGRIT	GSK1572932A	III	Peptide	MAGEA3	NSCLC	2278	Terminated	NCT00480025
N/A	CIMAvax-EGF	I/II	Protein-Protein Carrier Conjugate	EGF	NSCLC, Squamous Head and Neck	242	Suspended	NCT02955290
START	Tecemotide (L-BLP25)	III	Peptide encapsulated liposome	MUC1	NSCLC	1513	Completed	NCT00409188
START2	Tecemotide (L-BLP25)	III	Peptide encapsulated liposome	MUC1	NSCLC	35	Terminated	NCT02049151
TIME	TG4010	II/III	Modified, attenuated Ankara virus with IL-2	MUC1	NSCLC	148	Completed	NCT00415818
STOP	Belagenpumatuc el-L (Lucanix)	III	Irradiated allogeneic NSCLC cells	TGF1/2	NSCLC	532	Completed	NCT00676507
N/A	GVAX^®^ (CG8123)	II	Autologous irradiated tumor cells with GM-CSF adjuvant	Personalized	NSCLC	19	Terminated	NCT00074295

Note: N/A—not available.

**Table 2.** Current clinical attempts in lung cancer vaccinology.

Clinical Trial Name	Vaccine Name	Phase	Formulation	Antigen	Cancer Types	Enrollment	Status	ClinicalTrials.gov ID
N/A	NEO-PV-01	I	Peptide with polyICLC adjuvant	Up to 20 personalized neoantigens	NSCLC	38	Completed	NCT03380871
N/A	PANDA-VAC	I	Dynamically adjusted peptide with polyICLC adjuvant	Up to 6 persoanlized neoantigens	NSCLC, Squamous Head and Neck	6	Not yet recruiting	NCT04266730
N/A	N/A	I/II	Peptide	MUC1	NSCLC	30	Recruiting	NCT01720836
N/A	STEMVAC	II	plasmid DNA	CD105, Yb-1, SOX2, CDH3, MDM2	NSCLC	40	Recruiting	NCT05242965
EMPOWER VAX Lung 1	BNT116	II	uridine RNA-based lipopolyplex	MAGEA3, CLDN6, KK-LC-1, PRAME, MAGEA4, MAGEC1	NSCLC	100	Recruiting	NCT05557591
KEYNOTE-942	INTerpath-002	III	mRNA-LNP	Up to 34 personalized neoantigen	NSCLC	868	Recruiting	NCT06077760
N/A	Autogene Cevumeran (R07198457)	I	mRNA lipoplex	Up to 20 personalized neoantigens	Advanced or Metastatic Tumors	272	Active, not recruiting	NCT03289962
N/A	SLATEv1	I/II	Chimp Adenovirus and self-replicating RNA	20 shared neoantigens derived from commons KRAS and TRP53 mutations	Advanced Solid Tumor	39	Completed	NCT03953235
N/A	SLATE-KRAS	I	Chimp Adenovirus and self-replicating RNA in combination with autologous T cells reactive against KRAS mutations	4 repeats of the 4 most highly prevalent KRAS neoantigens	Metastatic Cancer	210	Recruiting	NCT06253520
N/A	MIDRIXNEO-LUNG	I	mRNA loaded monocyte-derived, type 1 DC	Personalized Neoantigens	NSCLC	6	Completed	NCT04078269

Note: N/A—not available.

**Table 3.** Advantages and Limitations of Current Vaccine Delivery Platforms.

Current Cancer Vaccine Technologies Under Development	Advantages	Limitations
*Lipid Nanoparticles (LNP)*		
Passive LNP Targeting	Enhanced retention in tumor tissues	Accelerated blood clearance of vaccine
	Enhanced antigen presentation through dendritic cell targeting	Accumulation of vaccine particles in liver
	Potential for targeted tissue or cellular delivery	Tumor microenvironment penetrance ability is unknown
Active LNP Targeting	Enhanced retention in tumor tissues	Accelerated blood clearance of vaccine
	Enhanced antigen presentation through dendritic cell targeting	Accumulation of vaccine particles in liver
	Highly specific tissue or cellular delivery	Tumor microenvironment penetrance ability is unknown
*RNA*		
Self-replicating RNA (srRNA)	*In situ* amplification of tumor antigen load	Not as effective at priming immune responses
	Effective to boost pre-existing immunity	Complex packaging process
	Sustained mRNA production *in situ*	
Circulating RNA (circRNA)	Greater stability than current linear mRNA vaccines	Complex packaging process
	More immune quiescent than current linear mRNA vaccines	Limited human efficacy studies
*Mucosal Vaccination*	Selectively boosts local lung anti-tumor immunity	Limited delivery approaches
	Shows superior enhancement of resident memory T and B populations	Limited pre-clinical data in cancer setting
